# Recent Studies on the Construction of MOF-Based Composites and Their Applications in Photocatalytic Hydrogen Evolution

**DOI:** 10.3390/molecules30132755

**Published:** 2025-06-26

**Authors:** Quanmei Zhou, Yuchen Wei, Yifan Liao, Jiayi Meng, Yamei Huang, Xinglin Wang, Huihui Zhang, Weilin Dai

**Affiliations:** State Key Laboratory of Porous Materials for Separation and Conversion, Shanghai Key Laboratory of Molecular Catalysis and Innovative Materials, Department of Chemistry, Fudan University, Shanghai 200438, China; 24110220121@m.fudan.edu.cn (Q.Z.); 24110220083@m.fudan.edu.cn (Y.W.); 23110220051@m.fudan.edu.cn (Y.L.); 23110220061@m.fudan.edu.cn (J.M.); 22110220028@m.fudan.edu.cn (Y.H.); 21110220106@m.fudan.edu.cn (X.W.); 20110220111@fudan.edu.cn (H.Z.)

**Keywords:** photocatalysis, MOF, MOF-based composites, semiconductors, plasmonic metals, photocatalytic hydrogen evolution

## Abstract

The development of metal–organic framework (MOF)-based composites for photocatalytic hydrogen evolution has garnered significant attention due to their tunable structures, high surface area, and abundant active sites. Recent advancements focus on enhancing light absorption, charge separation, and catalytic efficiency through strategies such as ligand functionalization, metal doping, heterojunction formation, and plasmonic coupling effects. For instance, modifications with Ir (III) complexes and Pt nanoparticles have significantly improved hydrogen evolution rates, while sandwich-structured MOF composites demonstrate optimized charge separation through tailored micro-environments and proton reduction efficiency. Additionally, integrating MOFs with semiconductors (e.g., CdS, g-C_3_N_4_) or plasmonic metals (e.g., Au) enhances visible-light responsiveness and stability. This review highlights key design principles, performance metrics, and mechanistic insights, providing a roadmap for future research in MOF-based photocatalysts for sustainable hydrogen production. Challenges such as long-term stability and scalable synthesis are also discussed to guide further innovations in this field.

## 1. Introduction

The accelerated pace of industrial expansion worldwide has precipitated dual challenges of energy scarcity and environmental degradation. The increasing consumption of fossil fuels has further exacerbated environmental pollution, and traditional fossil energy sources are gradually failing to meet the developmental demands of the current era [1]. Therefore, the development of clean and sustainable energy sources has become an urgent priority [2,3]. Hydrogen, as an ideal fuel, produces only water upon combustion, causing no further environmental pollution and making it a highly regarded clean energy source. Currently, photocatalysis has been successfully applied in various fields, including pollutant degradation [4], tumor therapy [5], CO_2_ reduction [6], and hydrogen production via water splitting [7]. Among these applications, photocatalytic hydrogen production technology has attracted significant attention. This technology is based on the principles of photocatalysis, utilizing semiconductor materials to absorb photon energy and generate photoinduced charge carriers (electron-hole pairs) and thereby efficiently converting solar energy into chemical energy stored in hydrogen and achieving truly green hydrogen production.

Semiconductor materials have garnered widespread attention in the field of photocatalytic hydrogen production. Materials such as CdS [8], TiO_2_ [9], C_3_N_4_ [10], and MoS_2_ [11] have been extensively studied for this purpose. However, despite their broad application, semiconductor materials often suffer from issues such as high charge carrier recombination rates and slow migration of photoinduced electrons, which severely hinder the efficiency of photocatalytic hydrogen production [12]. Current research focuses on MOF-derived photocatalytic materials, primarily due to their superior performance in photocatalytic reactions. MOF materials allow for precise control over morphology during preparation, enabling the optimization of structural characteristics to enhance charge carrier migration rates. Additionally, MOF materials exhibit excellent light absorption capabilities, and through appropriate modifications, their solar light utilization range can be broadened, allowing for more efficient harnessing of solar energy [13]. Most importantly, MOF materials possess high design flexibility. By selecting different metal ions and organic ligands, MOF-derived materials with specific functions and properties can be synthesized. For instance, introducing metal ions or organic groups with high photocatalytic activity can optimize the electronic structure of the material, improving the migration and separation efficiency of photoinduced charge carriers and thereby enhancing photocatalytic hydrogen production performance [14,15]. MOF-derived materials demonstrate unique advantages and broad research prospects in the field of photocatalytic hydrogen production.

This review systematically outlines the technical background of photocatalytic hydrogen production, providing an in-depth analysis of the current research status and challenges in photocatalytic technology. Focusing on the unique advantages of MOF-derived materials in photocatalytic hydrogen production, it elaborates on various optimization strategies, such as elemental doping, structural regulation, and morphological control, to enhance the hydrogen production capacity of photocatalytic materials. Furthermore, it comprehensively explores recent advancements in MOF-derived materials for photocatalytic hydrogen production, aiming to identify the key factors limiting the improvement of photocatalytic hydrogen evolution performance and provide insights for addressing the challenge of low efficiency. Through a comprehensive analysis of cutting-edge research in this field, this review aims to offer innovative ideas for developing more efficient photothermal technologies, thereby advancing photocatalytic hydrogen production to a new stage and laying the foundation for the large-scale sustainable production of clean energy.

## 2. Mechanism of Photocatalytic Hydrogen Evolution

**Figure 1:**
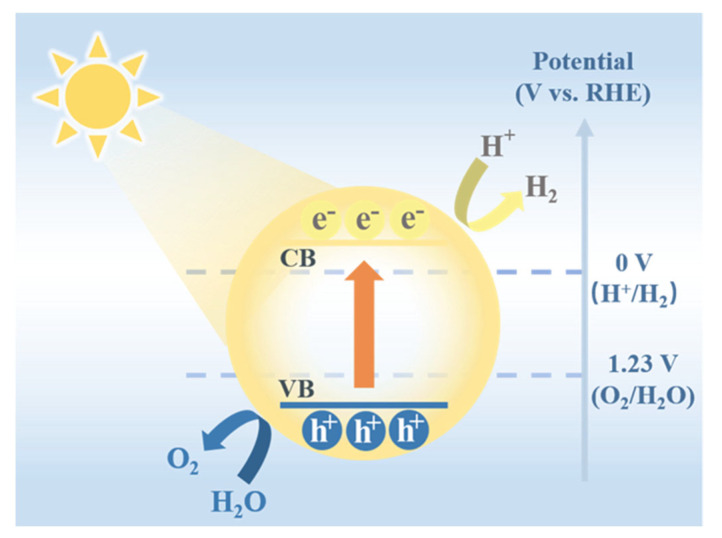
The mechanism of photocatalytic hydrogen evolution.

According to the second law of thermodynamics, the reaction of water splitting into hydrogen and oxygen (2H_2_O → 2H_2_ + O_2_) is an endergonic process; it requires external energy to drive the reaction (Endergonic refers to processes that require an input of free energy (i.e., ΔG > 0), as opposed to exergonic reactions that proceed spontaneously. While endothermic specifically denotes reactions that absorb heat (ΔH > 0), endergonic describes thermodynamic unfavorability in terms of Gibbs free energy, which depends on both enthalpy and entropy changes. Among various energy sources, solar energy stands out due to its renewability, sustainability, and nearly infinite reserves, making it a prime potential energy input for photocatalytic water splitting to produce hydrogen [2]. The process of hydrogen generation through photocatalytic water decomposition generally occurs via three consecutive stages, as illustrated in Figure 1.

The first step of photocatalysis is the light absorption and carrier excitation process. When a semiconductor photocatalyst is irradiated by sunlight, electrons in the valence band (VB) can absorb photon energy and transition to the conduction band (CB), forming photogenerated carriers [3]. This excitation process occurs only if the incident photon energy is greater than or equal to the semiconductor’s bandgap width. The second step involves the separation and migration of the generated photogenerated carriers. Upon the formation of photogenerated electron-hole pairs, electrons migrate to the conduction band, while holes remain stationary in the valence band. Subsequently, electrons move from the conduction band to the catalyst surface, where they participate in hydrogen evolution reactions, and holes are available at the valence band to oxidize sacrificial agents or other reactants, thus facilitating the overall photocatalytic hydrogen production process. However, due to the Coulomb interaction between electrons and holes, they tend to recombine to form a stable state, a process known as carrier recombination. The third step involves electrons and holes participating in redox reactions on the catalyst surface. Photogenerated electrons have reducing properties and can reduce protons (H^+^) adsorbed on the catalyst surface to hydrogen gas (H_2_). Photogenerated holes, conversely, have oxidizing properties and can oxidize water to oxygen (O_2_), or participate in other oxidation reactions, such as the oxidative degradation of organic pollutants. It is evident that the separation efficiency and recombination rate of photogenerated carriers are key factors limiting the performance of photocatalytic hydrogen production. To improve catalytic efficiency, researchers often construct heterojunctions and introduce cocatalysts to effectively suppress carrier recombination and promote their efficient separation and migration [4].

So far, the main indicators used to evaluate the photocatalytic performance of catalysts are apparent quantum efficiency (AQE) and solar-to-hydrogen energy conversion efficiency (STH). AQE is an important indicator to measure the efficiency of photocatalytic reactions [5]. In the process of photocatalytic reaction, a higher AQE value indicates better hydrogen production performance of the catalyst, indicating the ability of photon energy to drive the conversion of chemical reactants into products [16]:

(1) $$\mathrm{AQE}=\frac{2N{(H}_{2})}{N(photons)}$$

STH refers to the efficiency index of converting solar energy into hydrogen energy in the process of photocatalytic decomposition of water to produce hydrogen. It is expressed as the ratio of hydrogen energy produced by the hydrogen production system to the energy of incident light:

(2) $$\mathrm{STH}=\frac{output\;energy\;as\;H_{2}\;gas}{energy\;of\;incident\;solar\;light}$$

## 3. Research Progress on Photocatalytic Hydrogen Production of MOF Composites Modified Using Binary Materials

Metal–organic framework (MOF) materials have emerged as a research hotspot in photocatalytic hydrogen production owing to their high specific surface area, tunable pore structure, and abundant active sites [17]. The performance of MOFs in hydrogen production is strongly influenced by multiple structural and electronic properties, including pore architecture, active site density, redox-active metals, chemical stability, light-harvesting capability, and dimensional morphology, with the synergistic optimization of these factors leading to enhanced catalytic efficiency. The pore size of MOFs critically influences hydrogen production performance by affecting gas diffusion, active site exposure, structural stability, and light harvesting, with optimized pore architectures (1–5 nm) enhancing catalytic efficiency through improved mass transport and charge separation. However, single-component MOFs often suffer from inherent limitations, such as high photogenerated carrier recombination rates and low visible-light utilization efficiency. Modifying MOFs with binary materials to construct composite systems can significantly enhance their photocatalytic performance. This perspective systematically reviews the recent advances in binary material-modified MOF composites for photocatalytic hydrogen production, focusing on the construction strategies, performance enhancement mechanisms, and the latest research findings of semiconductor-MOF, metal nanoparticle-MOF, conductive polymer-MOF, and carbon material-MOF composite systems. The challenges and future development directions in this field are also discussed.

### 3.1. Semiconductor-MOF Composite System

Integrating semiconductor components with MOF matrices constitutes a viable approach to boosting the efficiency of light-driven hydrogen generation. Semiconductor materials typically exhibit excellent optoelectronic properties, and their combination with MOFs can form heterojunction structures that facilitate the separation and transport of photogenerated carriers [18]. Specifically, the following categories of semiconductor materials have been most extensively studied in MOF composite systems: metal oxides (e.g., TiO_2_, ZnO), metal sulfides (e.g., CdS, MoS_2_), and bismuth-based semiconductors (e.g., BiOBr, BiVO_4_).

The TiO_2_-MOF composite system has garnered significant attention due to its high stability and low cost. Jiang et al. innovatively grew TiO_2_ within the MIL-101 pores, constructing a “molecular compartment” structure that enabled the synergistic effect between the TiO_2_ light absorption unit and the MOF catalytic metal clusters [19]. This TiO_2_-in-MOF composite achieves an apparent quantum efficiency of 11.3% for CO_2_ reduction under 350 nm illumination, demonstrating excellent stability. Similarly, a collaborative team employed crystal phase engineering to construct a titanium-based material featuring the COK/MIL-125 heterostructure from 1,4-benzenedicarboxylic acid. The Z-type heterojunction at the interface significantly enhances photogenerated carrier separation efficiency, enabling the composite to achieve optimal hydrogen production under full-spectrum radiation. Further ligand modification with amino groups (NH_2_) extends light absorption into the visible spectrum, boosting visible-light-driven hydrogen production performance.

**Figure 2:**
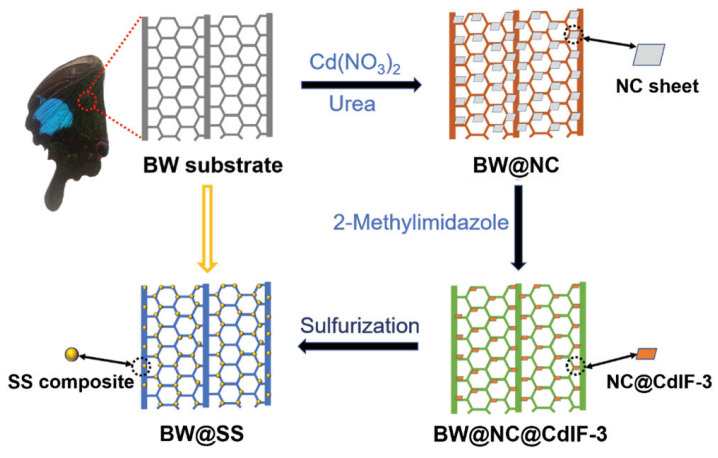
Illustration of the fabrication of BW@NC, BW@NC@CdIF-3, and BW@SS with reticular hierarchical structure [21]. Copyright 2022, Wiley.

Turning to metal sulfide-MOF composite systems, their narrow-bandgap characteristics effectively expand the light-responsive range [20]. Zhong and Liu reported the fabrication of hierarchical CdS/Cd_2_SO_4_(OH)_2_ (SS) composites using Papilio Paris butterfly wings as scaffolds combined with a MOF template-directed strategy (Figure 2) [21]. The process involved: first depositing cadmium carbonate (NC) doped with cadmium nitrate on butterfly wings to form BW@NC; then growing cadmium-based MOF (CdIF-3) on BW@NC to create reticular hierarchical BW@NC@CdIF-3; finally, using this as a sacrificial template to prepare butterfly wing@SS via sulfidation (BW@NC@CdIF-3 is a multi-layer composite material consisting of biochar (BW), nitrogen-doped carbon (NC), and a cadmium-based MOF (CdIF-3)). The unique structure of this material endows it with excellent photocatalytic performance, particularly in visible-light-driven hydrogen production reactions. Wing@SS refers to a functionalized material with wing-like nanostructures deposited on a stainless steel (SS) substrate to enhance its specific physical or chemical properties. Notably, MOF metal nodes linked by organic ligands endow the material with abundant exposed metal sites and prevent aggregation during sulfidation. The butterfly wing’s reticular hierarchical scaffold further enhances light absorption, improving photocatalytic performance. Meanwhile, doped Cd_2_SO_4_(OH)_2_ reduces to metallic cadmium, acting as electron traps to enhance charge separation. The composite’s photocatalytic activity and stability were verified via visible-light-driven hydrogen evolution reactions. In another investigation, BiOBr combined with NH_2_-Fe-MOF formed a 3D flower-like microsphere structure with a Type II heterojunction, increasing active sites, enhancing visible-light utilization, and promoting electron-hole pair separation. This composite achieved a 98.2% degradation rate for Rhodamine B within 40 min under visible light, with a rate constant k of 0.08507 min^−1^.

Bismuth-based semiconductor-MOF composite systems also exhibit promising application prospects. Bismuth-based semiconductors like BiVO_4_ and Bi_2_WO_6_ possess suitable band structures and visible-light response capabilities. Their combination with MOFs enables the formation of efficient carrier transport channels. For instance, BiOBr/NH_2_-Fe-MOF composites not only enhance the photocatalytic degradation performance of organic pollutants but also offer new insights for the development of photocatalytic hydrogen production materials.

The performance enhancement mechanisms of semiconductor-MOF composites can be systematically categorized as follows: (1) heterojunction formation promotes photogenerated carrier separation; (2) the extension of the light response range to the visible spectrum; (3) an increase in the number and accessibility of active sites; and (4) an improvement in photostability and cyclic performance. Future research should focus on exploring the combination of novel semiconductor materials with MOFs, optimizing interface engineering, enhancing charge separation efficiency, and deepening the understanding of structure–property relationships.

### 3.2. Metal Nanoparticles-MOF Composite System

Hybrid systems incorporating metallic nanoscale particles within MOF frameworks offer an alternative pathway for augmenting the performance of photocatalytic hydrogen evolution. The influence of different metal centers in various MOFs on hydrogen evolution can be attributed to several key factors, including the electronic structure, redox potential, coordination geometry, and intrinsic catalytic activity of the metal ions. This strategy can be systematically categorized into two major categories based on the metal type: noble metal nanoparticles (e.g., Pt, Pd, Au) and non-noble metal nanoparticles (e.g., Ni, Co, Cu). Noble metal NPs, owing to their surface plasmon resonance (SPR) effect and excellent electron capture capability, act as cocatalysts to significantly boost the photocatalytic activity of MOFs [22,23,24]. Non-noble metal NPs, conversely, have gained substantial attention due to their cost advantages.

Pd-modified MOF systems have demonstrated exceptional performance in photocatalytic hydrogen production [25,26,27]. For example, Kim et al. constructed 2D Pd-TCPP-based MOF nanosheets as single-atom photocatalysts (Pd-MOF), achieving a remarkable H_2_ production rate of 21.3 mmol·g^−1^·h^−1^—3.2 times higher than that of the analogous Pt-MOF (6.64 mmol·g^−1^·h^−1^) with similar noble metal loading [28]. This superior activity stems from the prolonged lifetime of photogenerated charge carriers in Pd-MOF (62 μs vs. 4 μs in Pt-MOF), enabled by weaker spin-orbit coupling in Pd-porphyrin, which facilitates efficient electron transfer to catalytic sites. Characterizations confirmed identical structural frameworks and light absorption for Pd-MOF and Pt-MOF, ruling out morphological or optical property differences. This study reveals that single-atom Pd embedded in 2D MOFs outperforms Pt counterparts via extended exciton lifetimes, challenging conventional wisdom and offering a new design paradigm for high-efficiency photocatalysts.

Shifting focus to Pd nanoparticle-MOF composites, their unique advantages have been increasingly recognized [29]. Pascanu et al. encapsulated bare Pd nanoparticles in MIL-101-NH_2_ to obtain Pd@MIL-101-NH_2_ [30], followed by post-synthesis modification to introduce hydrophobic perfluoroalkyl groups, yielding Pd@MIL-101-Fx (x = 3, 5, 7, 11, 15). This design enables the engineering of MOF pore walls to regulate the micro-environment around Pd and its substrate interactions. Results show that hydrophobic modification significantly improves activity and selectivity for both organosilane dehydrocoupling and halogenated nitrobenzene hydrogenation, attributed to enhanced substrate enrichment and regulated Pd-MOF host interactions [31].

**Figure 3:**
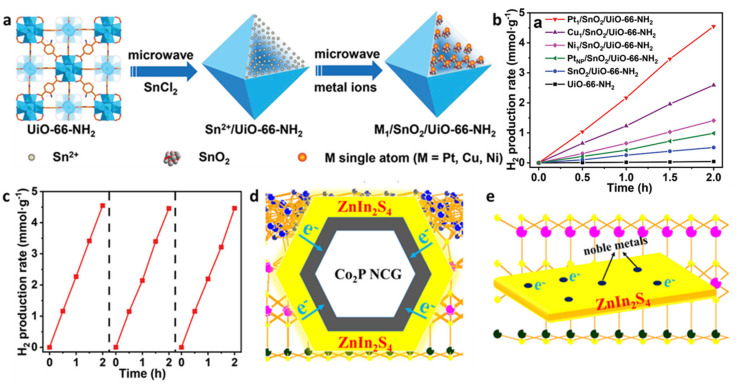
(**a**) Schematic illustration showing the microwave-assisted synthetic process for M_1_/SnO_2_/MOF. (**b**) Photocatalytic hydrogen production rates for Pt_1_/SnO_2_/UiO-66-NH_2_, Cu_1_/SnO_2_/UiO-66-NH_2_, Ni_1_/SnO_2_/UiO-66-NH_2_, PtNP/SnO_2_/UiO-66-NH_2_, SnO_2_/UiO-66-NH_2_, and UiO-66-NH_2_. (**c**) Performance in three consecutive runs of photocatalytic recycling for Pt_1_/SnO_2_/UiO-66-NH_2_, [32] Copyright 2021, Wiley. (**d**) The diffused electron separation over framework of Co_2_P/ZIS NCGs. (**e**) Limited active sites over noble metal loaded ZIS samples [33]. Copyright 2021, Elsevier.

Advancing to the atomic scale, the integration of single-atom catalysts (SACs) with MOFs has emerged as a frontier research area. Jiang et al. developed a universal strategy to immobilize SACs (Pt, Cu, Ni) in MOFs using SnO_2_ as a mediator, forming M1/SnO_2_/MOF architectures (Figure 3a) [32]. Microwave-assisted synthesis of SnO_2_-integrated MOFs (e.g., UiO-66-NH_2_, PCN-222, DUT-67) yielded Pt_1_/SnO_2_/UiO-66-NH_2_ with an outstanding hydrogen evolution rate of 2167 μmol·g^−1^·h^−1^—5 times higher than that of Pt nanoparticles on SnO_2_/UiO-66-NH_2_ and superior to Cu/Ni counterparts (Figure 3b,c) (UiO stands for University of Oslo, and UiO-66-NH_2_ is a specific MOF developed at the University of Oslo. These materials are known for their large surface areas, tunable pore structures, and functionalities, making them promising candidates for photocatalytic water splitting for hydrogen generation. The designation “66” serves as an identifier for a particular member within the UiO series of MOFs, highlighting its unique crystal structure and chemical composition. PCN-222 refers to porphyrinic MOF-222, which is a type of MOF modified with porphyrin groups. This material is widely used in various catalytic reactions due to its excellent photocatalytic performance. DUT-67 stands for Dresden University of Technology-67, a MOF developed at the Dresden University of Technology. This material features unique pore structures and large surface areas, demonstrating significant potential in gas adsorption and separation applications.). Characterizations confirmed atomically dispersed Pt sites with Pt-O_4_ coordination, while DFT calculations showed Pt single atoms exhibit the lowest hydrogen binding free energy (−0.28 eV), enabling efficient proton reduction. This work represents the first universal method to stabilize SACs with uniform micro-environments across diverse MOFs, highlighting SnO_2_-MOF synergy for enhanced photocatalysis.

To address noble metal dependency in photocatalytic hydrogen evolution (PHE), our group developed hierarchical Co_2_P/ZnIn_2_S_4_ nanocages (Co_2_P/ZIS NCGs). Calcination and phosphidation of ZIF-67 formed hollow Co_2_P nanocages, which were hydrothermally coated with ZnIn_2_S_4_ thin layers [33]. The resulting composite exhibited a hydrogen evolution rate of 7.93 mmol·g^−1^·h^−1^—10 times higher than that of pristine ZnIn_2_S_4_ and superior to 1% noble metal (Pt, Au, Ag)-loaded ZnIn_2_S_4_ catalysts. As shown in Figure 3d,e, the unique hollow structure of Co_2_P NCGs promotes photogenerated charge separation and transfer from ZnIn_2_S_4_ to Co_2_P, as confirmed through DFT calculations, enabling efficient noble metal-free PHE [34].

Performance optimization of metal nanoparticle-MOF composites requires consideration of key factors: (1) metal nanoparticle size, dispersion, and loading; (2) MOF pore structure confinement effects; (3) metal-MOF electronic interactions [35]; and (4) reaction micro-environment regulation (e.g., hydrophobicity, functional groups). Future research should focus on developing precise nanoparticle loading methods, deepening the understanding of metal-support interaction mechanisms, and exploring non-noble metal alternatives to reduce costs.

### 3.3. Metal Sulfides and Carbon Material-MOF Composite System

The combination of metal sulfides and carbon materials with MOFs provides a novel material design paradigm for photocatalytic hydrogen production. Such composites typically feature excellent charge transport, tunable band structures, and robust stability, effectively addressing the poor conductivity and high photogenerated carrier recombination rates inherent in pure MOFs.

**Figure 4:**
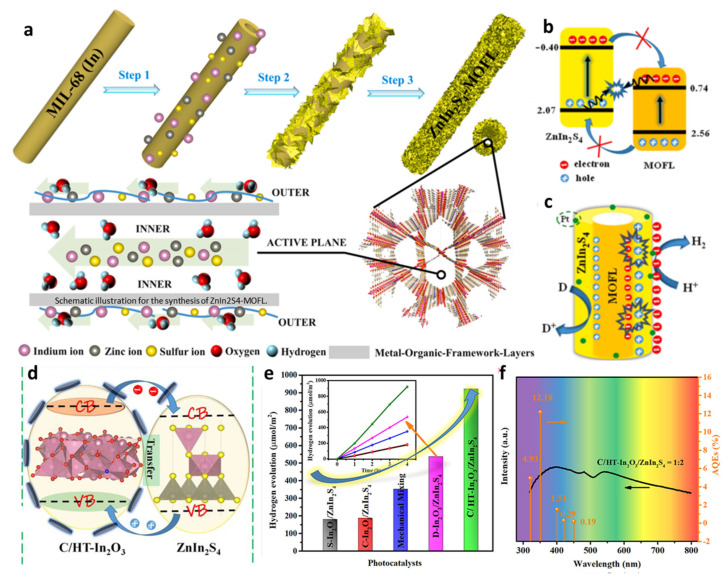
(**a**–**c**) Schematic illustration of the In−N−In sites in the interface of the carbon-coated HT-In_2_O_3_/ZnIn_2_S_4_ heterostructure for boosting charge transfer Copyright © 2021 American Chemical Society [36]. Copyright 2021, Elsevier. (**d**) Illustration of the formation mechanism of staggered C/HT-In_2_O_3_/ZnIn_2_S_4_ heterostructure and possibly smooth interfacial charge-transfer pathways. (**e**) H_2_ generation activity of comparative samples. (**f**) Apparent quantum efficiencies under 320, 350, 400, 420, and 450 nm at monochromatic light. [37] Copyright 2021, American Chemical Society.

Metal sulfide-MOF composite systems enhance photocatalytic performance through multiple mechanisms. For example, our group reported a facile one-step strategy to construct hollow tubular ZnIn_2_S_4_ modified by MOF-layers (MOFL) using In-MOF as a precursor (Figure 4a) [36]. This composite served as an efficient photocatalyst for robust photocatalytic hydrogen evolution, exhibiting an extraordinarily high rate of 28.2 mmol·g^−1^·h^−1^—approximately 14.8 times higher than that of pristine ZnIn_2_S_4_. The apparent quantum efficiency (AQE) reached 22.67% at 350 nm, and it maintained stable hydrogen generation (5.7 mmol·g^−1^·h^−1^) under real sunlight. As shown in Figure 4b,c, the innovation lies in constructing a direct Z-scheme system by integrating ZnIn_2_S_4_ with MOFL, which synergistically promotes charge separation. This system shows suppressed carrier recombination and a unique hollow tubular structure that enhances active site exposure and light absorption.

The mechanisms of metal sulfide-modified MOFs include the following: (1) extension of light absorption into the visible spectrum; (2) acting as electron transport channels to facilitate charge separation; (3) providing additional active sites; and (4) altering surface properties to optimize substrate adsorption. Notably, the method of introducing metal sulfides (e.g., in situ polymerization, post-modification) and their loading significantly impact composite performance, necessitating precise control for optimal results.

Let us turn to carbon material-MOF composite systems. Porous carbon materials like graphene [38], carbon nanotubes [39], and g-C_3_N_4_ [40] have been widely explored. Graphene or carbon nanotubes integrated with MOFs offer key advantages: (1) the high electrical conductivity of carbon materials promotes charge transfer; (2) a large specific surface area provides more active sites; (3) a strong π-π conjugated structure interaction between carbon materials and MOF organic ligands; and (4) robust heterojunction structures to promote photogenerated carrier separation.

Graphene/MOF composites are among the most extensively studied systems. The high electrical conductivity and excellent mechanical properties of graphene make it an ideal carrier or composite component for MOFs. Composites with three-dimensional conductive network structures can be prepared by growing MOF nanocrystals on graphene surfaces or reducing graphene oxide mixed with MOF precursors [41]. This strategy offers dual advantages: conductive carbon networks facilitate rapid charge migration, while porous MOF frameworks provide abundant active sites and structural stability. For example, in the C/HT-In_2_O_3_/ZnIn_2_S_4_ system (Figure 4d), carbon-coated hollow tubular In_2_O_3_ derived from In-MOF not only enhances charge separation via its conductive carbon layer but also forms a staggered heterojunction with ZnIn_2_S_4_, demonstrating a 13.2-fold increase in hydrogen evolution rate compared to pristine In_2_O_3_ (Figure 4e,f) [37]. Such synergistic integration of carbon and MOF materials represents an effective approach to boost photocatalytic performance for sustainable hydrogen production.

g-C_3_N_4_/MOF composites also show promising prospects [42]. g-C_3_N_4_, a typical polymer semiconductor with appropriate band structure and good chemical stability, forms Type II or Z-type heterojunctions with MOFs, effectively promoting photogenerated electron–hole pair separation. Moreover, the nitrogen-rich characteristics of g-C_3_N_4_ synergize with MOF metal nodes, thereby optimizing the electronic structure of catalytic active sites.

Future development of metal sulfide and carbon material-MOF composite systems should focus on the following: (1) developing precise composite methods to control interfacial structures and interactions; (2) exploring new metal sulfides and carbon materials (e.g., conjugated microporous polymers, carbon quantum dots) for MOF composites; (3) investigating mechanisms through which external fields (e.g., pressure, electric fields) affect composite performance; and (4) optimizing material design for efficient solar-to-chemical energy conversion.

## 4. Research Progress on Ternary Composites Based on MOF in Photocatalytic Hydrogen Production

MOF materials have become a focal point in photocatalytic hydrogen production research, attributed to their prominent features including high specific surface area, adjustable pore architecture, and plentiful active sites. Nevertheless, standalone MOF materials frequently encounter challenges like rapid recombination of photogenerated carriers and limited light response spectra. The fabrication of three-component systems markedly improves the catalytic activity of MOF-derived photocatalysts through cooperative interactions [43]. This review systematically examines the recent advances in MOF-based ternary composites for photocatalytic hydrogen production, focusing on material design strategies, performance enhancement mechanisms, and typical systems, including noble metal/semiconductor/MOF, quantum dot/MOF/cocatalyst, and multi-acid/MOF/semiconductor composite systems. The current challenges and future directions are also discussed to guide the design of efficient MOF-based photocatalysts.

MOFs are porous crystalline materials formed via the self-assembly of metal ions/clusters with organic ligands via coordination bonds. They feature ultra-high specific surface areas (typically 1000–10,000 m^2^/g), tunable pore structures, and abundant active sites, making them highly promising in gas adsorption, catalysis, sensing, etc. In photocatalysis, the periodic structure of MOFs facilitates photogenerated carrier transport, while functionalizable organic ligands enable modulation of light absorption. However, single-component MOFs often exhibit high photogenerated electron-hole recombination rates and limited light response (predominantly in the UV region), severely restricting improvements in their photocatalytic performance [44]. To address these limitations, researchers have developed various modification strategies, with constructing ternary composites emerging as an effective approach to enhance MOF-based photocatalytic hydrogen production. By organically combining MOFs with two other functional materials (e.g., semiconductors, noble metal nanoparticles, quantum dots, carbon materials), ternary composites achieve synergistic effects through multiple mechanisms: extending light response [45] (via narrow-bandgap semiconductors/photosensitizers to visible/near-infrared regions), promoting charge separation (via heterojunction/Z-scheme structures), providing additional active sites (via cocatalysts to reduce hydrogen evolution overpotential), and enhancing stability (via protective shells/frameworks to prevent photocorrosion/aggregation).

**Figure 5:**
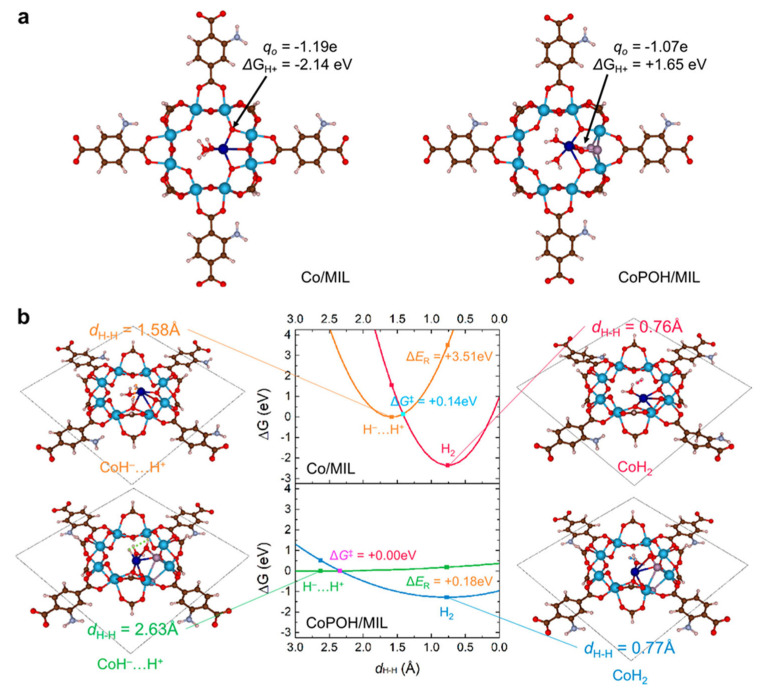
(**a**) Theoretical Gibbs free energy of protonation and the Marcus kinetics of Tafel processes of Co/MIL and CoPOH/MIL. Theoretically optimized MOF structures of Co/MIL and CoPOH/MIL. The numbers are the Bader charge of the oxygen atoms bonded to a Co atom and their proton binding free energies. (**b**) Gibbs free energy surfaces (ΔG in eV) of H_2_ bond formation in a Tafel process at Co/MIL and CoPOH/MIL based on Marcus kinetic theory of electron transfer [46]. Copyright 2024, American Chemical Society.

MOF-based ternary composites have shown significant advances in photocatalytic hydrogen production. For instance, Jung et al. designed a MOF photocatalyst with cooperative Brønsted acid–single-atom catalytic sites by grafting Co single atoms onto Ti-oxo clusters in MIL-125-NH_2_ and introducing P–OH moieties as Brønsted acid sites [46]. The optimized CoPOH/MIL exhibited a visible-light-driven H_2_ production rate of 6.6 mmol·g^−1^·h^−1^—6.6 times higher than that of Pt nanoparticles and three orders of magnitude higher than that of catalysts lacking Brønsted acid sites. As illustrated in Figure 5, DFT calculations revealed that the synergistic interaction between Co single atoms and adjacent Brønsted acid sites accelerates proton transfer and reduces the H_2_ evolution activation barrier, highlighting the importance of cooperative dual sites in enhancing photocatalytic efficiency.

### 4.1. Design Strategy and Construction Method of MOF-Based Ternary Composites

Constructing an efficient MOF-based ternary photocatalyst requires meticulous design of synergistic component interactions and the adoption of appropriate synthesis methods to ensure precise structural control. Based on component properties and functions, MOF-based ternary composites can be primarily categorized into distinct design strategies, each featuring unique advantages and suitable synthesis approaches.

**Figure 6:**
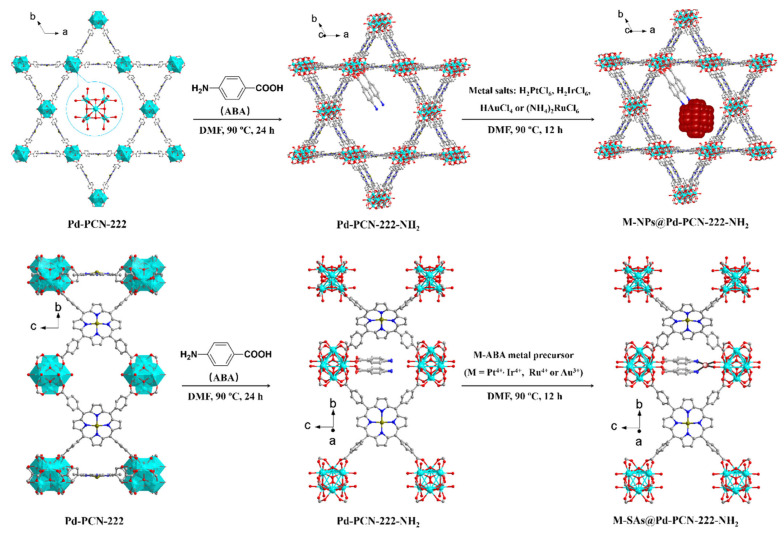
Schematic illustration showing the syntheses of M-NPs@Pd-PCN-222-NH_2_ with normal metal salt reduction and loading into the large pore-spaces (**upper**) and M-SAs@Pd-PCN-222-NH_2_ with the precoordination confinement strategy in the middle pore-spaces (**lower**) [47]. Copyright 2022, American Chemical Society.

The noble metal/MOF/semiconductor system represents a widely investigated ternary strategy. Here, components play defined roles: MOFs serve as the main framework, offering high specific surface area and ordered pore structures; semiconductors (e.g., TiO_2_, ZnO, CdS) handle light absorption and primary charge separation; noble metal nanoparticles (e.g., Pt, Au, Ag) act as electron traps and cocatalysts to reduce hydrogen evolution overpotential. For example, as shown in Figure 6, single-atom catalysts (M-SAs) spatially isolated in porphyrin MOF nanopores were prepared, yielding M-SAs@Pd-PCN-222-NH_2_ (M = Pt, Ir, Au, Ru) composites [47]. These exhibited exceptional durability in visible-light-driven (λ ≥ 420 nm) hydrogen evolution, achieving a turnover number (TON) of 21,713 within 32 h, with initial/sustained turnover frequencies (TOF) exceeding 1200/600 h^−1^. Photoelectrochemical analyses and DFT calculations revealed that chemically binding-stabilized integration of Pt-SAs with Pd-porphyrin photosensitizers accelerates electron-hole separation and charge transfer in pore nanospaces, persistently enhancing photocatalysis.

Another strategy is the quantum dot/MOF/catalyst system, leveraging quantum dot size effects to modulate band structures. Quantum dots (e.g., CdSe, CdTe, graphene QDs) offer tunable bandgaps and efficient carrier generation, while MOFs act as dispersing matrices to prevent QD aggregation.

The multi-acid/MOF/semiconductor system utilizes polyoxometalates (POMs) for electron storage/transfer. POMs with abundant redox sites capture photogenerated electrons and transfer them to protons. POM@MOF composites combine POM redox activity with MOFs’ high surface area, preventing POM aggregation and exposing more active centers. Adding semiconductors extends light response, forming efficient ternary systems.

### 4.2. Synthesis Method and Structure Control

In situ growth is a common method for constructing MOF-based ternary composites, where MOF frameworks grow around pre-synthesized nanoparticles to achieve uniform dispersion and close component contact. Researchers prepared a novel Au@PCN-222 catalyst via one-dimensional channel-confined growth of gold nanorods within MOFs [48]. This structure retains the high specific surface area and CO_2_ adsorption capacity of MOFs while enhancing photocatalytic performance through the plasmonic effect of Au nanorods. In situ growth is particularly suitable for constructing core-shell structured ternary composites. For example, our team constructed a 3D/2D ZnIn_2_S_4_/Ni-MOLs heterojunction via an in situ hydrothermal method, tightly anchoring 3D ZnIn_2_S_4_ nanoflowers onto 2D Ni-MOLs nanosheets [49]. The optimized composite exhibited an outstanding hydrogen evolution rate of 4.75 mmol·g^−1^·h^−1^ under visible light—8 times higher than that of pure ZnIn_2_S_4_ and 95 times higher than pure Ni-MOLs—with an apparent quantum efficiency (AQE) of 23.3% and excellent stability over five cycles. The in situ formed a tight interface, confirmed via in situ XPS and DFT calculations, promotes efficient electron transfer from ZnIn_2_S_4_ to Ni-MOLs, synergistically enhancing charge separation, visible-light absorption, and active site exposure.

Post-synthesis modification involves the sequential introduction of other functional components onto as-synthesized MOFs. This method is operationally simple and allows for flexible control over component loading. While post-modification enables independent optimization of each component’s synthesis conditions, it may suffer from uneven component distribution [50]. For instance, amino-functionalizing MOF pores before loading noble metal nanoparticles allows precise tuning of active site density.

**Figure 7:**
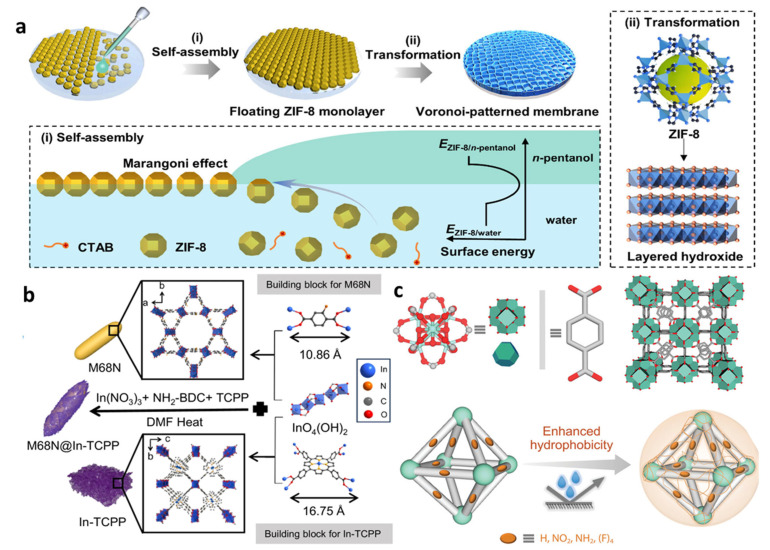
(**a**) Schematic illustrating the process: (i) self-assembly of ZIF-8 particle monolayers driven by the Marangoni effect; (ii) subsequent hydrolysis-induced transformation into Voronoi-CMs, [53] Copyright 2025, American Chemical Society. (**b**) Schematic representation for producing M68N, In-TCPP, and M68N@In-TCPP core@shell MOFs structures via the one-pot method, [54] Copyright 2025, Nature. (**c**) Schematic illustration of the adopted polymer (PDMS and/or OS) protection strategy, primed to induce surface hydrophobicity [55]. Copyright 2025, Wiley.

Self-assembly utilizes intermolecular forces (such as electrostatic and coordination interactions) to spontaneously form ternary composite structures (Figure 7a). Multinuclear acids@MOFs composites are often prepared using this method, where POMs spontaneously penetrate into the pores of MOFs through impregnation [51]. The self-assembly process is mild and allows for the preservation of the intrinsic properties of each component, but it requires a high degree of compatibility between components [52,53].

The key to constructing an efficient MOF-based ternary photocatalyst lies in optimizing the interfacial characteristics and band matching between components. Strong interface contact facilitates charge transfer, while appropriate band alignment drives the directional separation of photogenerated carriers [56].

Heterojunction design is an effective strategy for optimizing interface characteristics [57]. Gálvez-Barbosa et al. report that the dual Z-scheme iron-based MOF ternary composite material (Fe-based MOF/silver-containing compound/silver-containing compound) further enhances redox reaction capability due to the increased number of transport channels. Compared to single Z-type heterojunctions, the dual Z-type structure offers significant advantages in light capture and charge transfer [58].

Designing MOF-based ternary composites requires a comprehensive consideration of component selection, synthesis methods, and interface engineering. By rationally designing component functions and synergistic effects and employing appropriate synthesis strategies to control material structure, efficient and stable photocatalytic hydrogen production systems can be developed [59]. With advancements in characterization techniques and theoretical calculations, the understanding of material structure–property relationships will deepen, guiding the design of superior ternary composites.

For example, in the α-DMACoPc/TiO_2_/MIL-101 (Fe) ternary composite material, phthalocyanine molecules act as photosensitizers and coordinate with MOF [60,61] defect sites through carboxylic acid groups, forming stable interfacial connections. This chemical bonding not only enhances the stability of the composite material but also promotes electron transfer at the interface.

MOF-based ternary composites exhibit outstanding performance in photocatalytic hydrogen production through carefully designed component combinations and structural configurations. This section analyzes typical cases including noble metal-modified systems, quantum dot composites, and polyoxometalate hybrid systems [62], exploring their compositional characteristics, performance advantages, and action mechanisms.

The dual Z-scheme heterojunction enhances carrier separation efficiency through internal charge transport. MOF-based heterojunction is able to be constructed using the one-pot method (Figure 7b). Surface modification can also improve interfacial properties. By introducing bridging molecules or functional groups, the interaction between components can be enhanced, promoting charge transfer at the interface (Figure 7c).

### 4.3. Precious Metals/MOF/Semiconductors Ternary System

The iridium complex/Pt/UiO-66-NH_2_ system exemplifies noble metal-modified MOF composites. Deng et al. prepared the cmrIr/Pt@UiO-66-NH_2_ composite via post-synthetic modification [63], anchoring iridium (III) complexes with excellent light-absorbing properties onto the surface of Pt@UiO-66-NH_2_. (CmrIr refers to a cationic iridium (III) complex functionalized with coumarin-based ligands and carboxylate groups. This complex exhibits excellent light-absorbing properties and significantly enhances the visible-light-driven hydrogen production activity when integrated with MOF materials.) In this design, UiO-66-NH_2_ provides a stable porous framework and coordination sites, while platinum nanoparticles act as electron traps to facilitate proton reduction. The iridium complex extends light absorption to the visible spectrum through its coumarin ligands. Notably, even at an extremely low iridium content (0.2%), the hydrogen yield of this ternary composite reaches 446.4 μmol·g^−1^, approximately 2.5 times that of the binary Pt@UiO-66-NH_2_ system (180.7 μmol·g^−1^). Solid-state UV spectroscopy, photocurrent response, and electrochemical impedance tests confirm that the introduction of iridium (III) complexes significantly promotes electron–hole pair separation, thereby enhancing the overall photocatalytic activity.

Similarly, Cui et al. developed a Z-scheme MIL-53(Fe)/α-Bi_2_O_3_/g-C_3_N_4_ ternary photocatalyst that demonstrated superior visible-light-driven degradation of amino black 10B (optimal at 32% MIL-53(Fe) loading) through enhanced light absorption and efficient charge separation via a direct solid-state dual Z-scheme mechanism while maintaining excellent stability over four recycling runs. [64]. The dual Z-scheme heterojunction photocatalyst Ag/AgVO_3_/g-C_3_N_4_ was synthesized via a wet-impregnation method. Under visible-light irradiation, this catalyst demonstrates outstanding degradation performance for expired ciprofloxacin (CIP) and simultaneously enables hydrogen production from natural rainwater without the need for sacrificial reagents. Notably, its apparent quantum efficiency (AQE) reaches 9.95% at 420 nm, vividly illustrating the application potential of dual Z-scheme heterojunctions in the field of photocatalysis [65]. Compared to single Z-type heterojunctions, the dual Z-type structure, due to the increased number of transport channels, can simultaneously transfer electrons from the conduction band of semiconductor A to the valence band positions of semiconductors B and C, thus enabling more efficient redox reactions. This design concept provides a new direction for constructing highly efficient MOF-based tricatalyst materials.

### 4.4. Quantum Dot/MOF/Catalyst Ternary System

**Figure 8:**
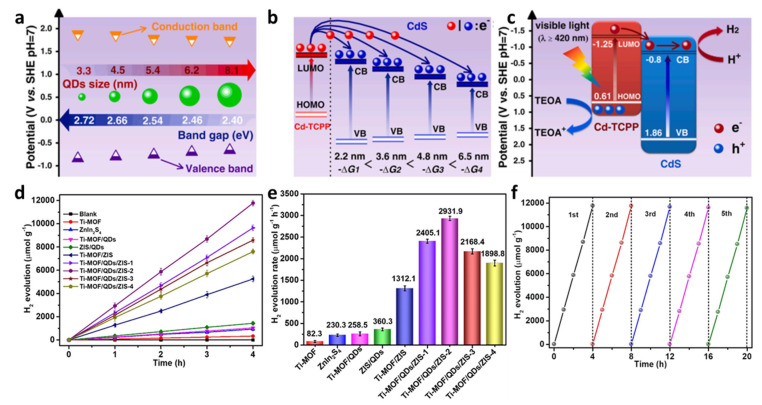
(**a**) Size-dependent bandgaps and energy-band positions of CdS QDs with different sizes. (**b**) Schematic diagram of the CB position dependence of −ΔG. The red and the blue spheres denote photoinduced electrons. (**c**) The excitons dissociation and transfer in the CdS/Cd-TCPP heterostructure under visible-light irradiation. The red and the blue spheres denote photoinduced electrons and holes [66]. Copyright 2022, Elsevier. (**d**) Photocatalytic H_2_ evolution versus time for the Ti-MOF/QDs/ZIS photocatalysts and reference photocatalysts under visible light irradiation, (**e**) corresponding photocatalytic H_2_ evolution rates, (**f**) Recycle experiments for the H_2_ generation over the Ti-MOF/QDs/ZIS-2 photocatalyst [67]. Copyright 2022, Elsevier.

Quantum dots (QDs) integrated with MOFs have emerged as efficient photocatalysts for hydrogen evolution, leveraging quantum confinement effects and MOF-derived structural advantages. Xu et al. constructed size-controlled CdS QDs (2.2–6.5 nm) on Cd-TCPP nanosheets via partial sulfidation, forming CdS/Cd-TCPP heterojunctions that exhibited a superior hydrogen evolution rate of 3150 μmol·g^−1^ h^−1^ (5-fold higher than that of pure CdS) under visible light [66]. The optimal performance of 4.8 nm CdS/Cd-TCPP originates from the synergistic effect of enhanced light absorption by Cd-TCPP and efficient electron injection from Cd-TCPP to CdS QDs, regulated via QD size-dependent conduction band alignment. In situ XPS and DFT calculations confirm that moderate QD size (4.8 nm) balances electron transfer efficiency and light harvesting, demonstrating that QD/MOF heterostructures with tailored interfacial charge dynamics offer a promising strategy for high-performance photocatalytic hydrogen production (Figure 8a–c).

Quantum dots (QDs) and MOFs frequently form Z-scheme heterojunctions to boost photocatalytic hydrogen production performance [67]. A recent study introduces a novel all-solid-state Z-scheme composite, NH_2_-MIL-125(Ti)/Ti_3_C_2_ MXene quantum dots (QDs)/ZnIn_2_S_4_ (Ti-MOF/QDs/ZIS), where 2D ZnIn_2_S_4_ nanosheets are grown on 3D Ti-MOF and anchored with 0D Ti_3_C_2_ QDs as electron mediators (Figure 8d–f) [67]. This architecture leverages the narrow bandgap of ZnIn_2_S_4_ (2.43 eV) for visible-light absorption, the high specific surface area of Ti-MOF (1063.5 m^2^/g), and the metallic conductivity of Ti_3_C_2_ QDs to facilitate rapid interfacial charge transfer. Under visible light, Ti-MOF/QDs/ZIS achieves exceptional hydrogen evolution rates of 2931.9 μmol·g^−1^·h^−1^, 12.7 times higher than that of pure ZnIn_2_S_4_, and degrades tetracycline (96% in 50 min) and sulfamethazine (98% in 40 min) via O_2_^−^-dominated pathways. The Z-scheme mechanism is confirmed through in situ XPS and photoelectrochemical tests, showing that Ti_3_C_2_ QDs bridge electron transfer from the conduction band of ZnIn_2_S_4_ to the valence band of Ti-MOF, preserving high-redox capability in both components. This design prevents charge recombination, as evidenced by reduced PL intensity and prolonged carrier lifetime (2.60 ns vs. 1.32 ns for pure ZnIn_2_S_4_). LC-MS analysis reveals intermediate degradation products of antibiotics, with toxicity assessment indicating reduced hazard during mineralization. The composite maintains 95% activity after five cycles, demonstrating stability crucial for practical applications. This work highlights MXene QDs as efficient noble metal-free electron mediators in Z-scheme systems, offering a scalable approach for integrated hydrogen production and wastewater treatment.

### 4.5. Polyacid/MOF/Semiconductor Ternary System

Polyoxometalate (POMs)@MOF composites integrate the excellent redox capabilities of POMs with the high specific surface area characteristics of MOFs. A research team has summarized that POM@MOF composites, owing to their ultra-high porosity, large specific surface area, and superior redox properties, represent a promising class of photocatalytic materials [68]. In these composites, the porous framework of MOFs ensures uniform dispersion of POMs in aqueous solutions, thereby enhancing the stability of POMs. Meanwhile, POMs possess abundant active sites and exceptional ability to capture light-excited electrons, effectively retarding the recombination of photogenerated carriers and improving photocatalytic efficiency.

Building on this foundation, POM@MOF/semiconductor ternary systems further expand the light response range by introducing semiconductor components. In this design, semiconductors (such as TiO_2_ and CdS) serve as primary light absorbers, POMs act as electron mediators to facilitate charge transfer, and MOFs provide ordered pore structures and high specific surface areas. Studies have shown that precisely structured POM@MOF composites not only leverage the advantages of both POMs and MOFs but also avoid their respective drawbacks. By reasonably selecting semiconductor components, efficient full-spectrum responsive photocatalytic hydrogen production systems can be constructed.

## 5. Summary and Outlook

This review has systematically examined recent advances in MOF-based composites for visible-light-driven hydrogen production. MOF-based composites have demonstrated significant progress in photocatalytic hydrogen production, with researchers successfully developing high-performance systems through construction methods including physical mixing, in situ growth, post-synthesis modification, templating, and self-assembly. These composites enhance photocatalytic hydrogen production efficiency and stability by optimizing interfacial contact, modulating band structures, expanding light absorption ranges, and improving surface reactivity. In-depth studies of their photocatalytic mechanisms provide critical theoretical support for material design and optimization. Table 1 shows the comparison of different MOF-based composites in photocatalytic hydrogen evolution reactions.

However, despite these advancements, MOF-based composites face challenges in practical applications: enhancing light absorption to improve solar energy utilization, improving long-term stability to meet industrial requirements, and addressing large-scale preparation and cost-control issues. Despite the vast number of reported MOFs, several types—including highly porous MOFs with tailored pore sizes, functionalized-linker MOFs, redox-active metal-centered MOFs, nanoparticle-integrated MOFs, and flexible MOFs—remain underexploited in hydrogen production due to limited exploration and challenges in stability and scalability.

Prospective investigations should concentrate on the following:

1. Novel composite design: developing new MOF-based composites (e.g., MOF-semiconductor, MOF-2D material, MOF-covalent organic framework (COF) systems) to achieve efficient light absorption and carrier separation.

2. Light absorption enhancement: broadening light absorption (especially in visible/near-infrared regions) via structural design and chemical modification.

3. Enhanced stability: develop more stable MOF-based composites through surface modification, doping, or constructing core-shell structures to improve the material’s resistance to agglomeration and photodegradation during photocatalytic processes.

4. In-depth mechanism research: utilize advanced characterization techniques and theoretical calculations to further elucidate the separation, transport, and surface reaction mechanisms of photogenerated carriers in MOF-based composites, providing more precise guidance for material optimization.

5. Application expansion and industrialization: explore the application of MOF-based composites in practical photocatalytic hydrogen production systems, addressing issues such as large-scale preparation, cost control, and system integration. To promote its commercial application from the laboratory. To advance MOFs toward commercialization, efforts should focus on scalable synthesis, stability enhancement, performance optimization, integration with industrial processes, addressing safety and regulatory issues, and promoting market awareness through education and collaboration.

In conclusion, MOF-based composites show broad prospects in photocatalytic hydrogen production. Continued research and technological advancements will drive the development of high-performance, low-cost, and stable MOF-based photocatalysts, supporting sustainable clean energy production.

## Figures and Tables

**Table 1:** Photocatalytic hydrogen evolution performance of MOFs based composites.

Catalyst	Synthesis Method	Cocatalyst/ Sacrificial Agent	H_2_ Evolution Rate	Light Source	Ref
In_2_O_3_/ZnIn_2_S_4_	Solvothermal	Pt/Na_2_S·Na_2_SO_3_	920.5 μmol·g^−1^·h^−1^	300 W Xe lamp	[37]
M-TBAPy (M: In Al Sc Ti)	Hydrothermal	TEA	147.5 μmol·g^−1^·h^−1^	300 W Xe lamp	[69]
ZnIn_2_S_4_-MOF	One-step method	Pt/Na_2_SO_4_	28.2 mmol·g^−1^·h^−1^	300 W Xe lamp	[36]
ZnIn_2_S_4_/Ni-MOLs	Hydrothermal	Pt NPs/TEOA	4.75 mmol·g^−1^·h^−1^	300 W Xe lamp	[49]
Au/Ni-MOF	Wet chemical method	Au/Na_2_SO_3_	7610 μmol·g^−1^·h^−1^	AM 1.5 G	[70]
EY-6Cu-NU-66	Impregnation strategy	EY/TEOA	3579.82 μmol·g^−1^·h^−1^	300 W Xe lamp	[71]
Pt/Zr-TCPP(Pd)	Bottom-up method	Pt/Ascorbic acid	3348 μmol·g^−1^·h^−1^	λ ≥ 400 nm	[58]
Ti/CeUiOMOFs@TiO_2_	Multistep strategy	TEOA	4724 μmol·g^−1^·h^−1^	UV	[72]
Bi_2_MoO_6_/Zn-TCPP	In situ self-assembly growth	Pt/Ascorbic acid	10,900.94 μmol·g^−1^·h^−1^	λ ≥ 420 nm	[73]
CoMoC/ZnIn_2_S_4_	Solvothermal	TEOA	2232 μmol·g^−1^·h^−1^	300 W Xe lamp	[74]
Pt/PdTCPP + UiO-66-(NH_2_)_2_	Mixed-ligand strategy	Pt NPs/TEOA	1152 μmol·g^−1^·h^−1^	300 W Xe lamp	[75]
B-CTF-Ti-MOF	Solvothermal	Pt/TEOA	1975 μmol·g^−1^·h^−1^	300 W Xe lamp	[76]
Pt@CoCuZn-PMCPs-2	Solvothermal	Pt-EY/TEOA	1038.8 μmol·g^−1^·h^−1^	λ > 420 nm	[77]
Cu-In-Zn-S/Ni-MOF	Hydrothermal	Ascorbic acid	2642 μmol·g^−1^·h^−1^	λ > 420 nm	[78]
MIL-125-NH_2_/Ni_2_P	Solvothermal	Ni_2_P/TEA	4327 μmol·g^−1^·h^−1^	300 W Xe lamp	[79]
UiO-66-NH_2_@Pt@UiO-66-H	Self-assemble	Pt NPs/TEA	2708.2 μmol·g^−1^·h^−1^	300 W Xe lamp	[80]
PMF/G-25	Solvothermal	EY/TEOA pH = 9	1688.5 μmol·g^−1^·h^−1^	Multichannel reaction system	[81]
CdS@N-NiCoO	Solvothermal	Na_2_S·Na_2_SO_3_	4632 μmol·g^−1^·h^−1^	300 W Xe lamp	[82]
CdS/UiO-67-NH_2_	One-step method	TEOA	487.5 μmol·g^−1^·h^−1^	Visible light	[83]
Pt/UiO-66-pz	Hydrothermal	Pt NPs/TEA	329 μmol·g^−1^·h^−1^	Visible light	[84]
PdS@UiOS@CZS	Solvothermal	Na_2_S·Na_2_SO_3_	460.8 μmol·g^−1^·h^−1^	300 W Xe lamp	[85]

## Data Availability

No new data were created or analyzed in this study.
